# In silico identification of A1 agonists and A2a inhibitors in pain based on molecular docking strategies and dynamics simulations

**DOI:** 10.1007/s11302-021-09808-4

**Published:** 2021-10-22

**Authors:** Guangya Xu, Shutao Zhang, Lulu Zheng, Zhongjiao Hu, Lijia Cheng, Lvlin Chen, Jun Li, Zheng Shi

**Affiliations:** 1 Clinical Genetics Laboratory, Clinical Medical College & Affiliated Hospital & College of Basic Medicine & College of Food and Biological Engineering, Chengdu University, Chengdu, 610081 China; 2 School of Pharmacy, Zunyi Medical University, Zunyi, 563000 China; 3 Sichuan Wuyan Biotech Co. Ltd Company, Chengdu, 610041 China

**Keywords:** Adenosine A1 receptor, Adenosine A2a receptor, Pain, Potential agents, Drug discovery

## Abstract

Most recently, the adenosine is considered as one of the most promising targets for treating pain, with few side effects. It exists in the central nervous system, and plays a key role in nociceptive afferent pathway. It is reported that the A1 receptor (A1R) could inhibit Ca^2+^ channels to reduce the pain like analgesic mechanism of morphine. And, A2a receptor (A2aR) was reported to enhance the accumulation of AMP (cAMP) and released peptides from sensory neurons, resulting in constitutive activation of pain. Much evidence showed that A1R and A2aR could be served as the interesting targets for the treatment of pain. Herein, virtual screening was utilized to identify the small molecule compounds towards A1R and A2aR, and top six molecules were considered as candidates via amber scores. The molecular dynamic (MD) simulations and molecular mechanics/generalized born surface area (MM/GBSA) were employed to further analyze the affinity and binding stability of the six molecules towards A1R and A2aR. Moreover, energy decomposition analysis showed significant residues in A1R and A2aR, including His1383, Phe1276, and Glu1277. It provided basics for discovery of novel agonists and antagonists. Finally, the agonists of A1R (ZINC19943625, ZINC13555217, and ZINC04698406) and inhibitors of A2aR (ZINC19370372, ZINC20176051, and ZINC57263068) were successfully recognized. Taken together, our discovered small molecules may serve as the promising candidate agents for future pain research.

## Introduction

The pain is a staggering and urgent widespread public health problem. There were almost one billion patients who persecuted by pain, and eight million human beings died of it by 2020 [1]. Furthermore, pain threatened one-fifth of the adults approximately around the world, and it often caused various disease states, traumas, and operations [2]. Nevertheless, the preclinical literature demonstrated that current painkillers, such as non-steroidal anti-inflammatory agents (NSAIDs), paracetamol, and weak opioids could bring potential side effects to patients, leading to kidney failure, stomach ulcer, and liver damage [3]. It is known to all that adenosine was consisted of adenine and pentose, spreading throughout the human body [4]. Based on various affinity of adenosines, the adenosine receptors were classified to certain types such as A1, A2, and A3 receptors [5]. Among them, A1 and A2a receptors were considered as the promising anti-pain targets [6].

Adenosine was a kind of non-selective natural agonist of adenosine receptors. Injecting intrathecal adenosine was employed to reduce allodynia, chronic pain, and hyperalgesia in patients via activating A1Rs [7]. However, it may lead to side effects with activation of A2aRs, such as waist pain and headache [8]. Activated A2aRs can produce peripheral sensitive pain and vasodilator [9]. Moreover, it was difficult to achieve the analgesic effects by activating A1Rs to reduce the level of cAMP [10, 11].

Previous studies indicated that A1R induced spinal cord anti-injury sensation through the interaction with PTX sensitive G protein, and inhibited adenylate cyclase to reduce cAMP level [12]. The high cAMP level would promote the release of neurotransmitters [13]. Meanwhile, it could enhance the excitability of dorsal horn neurons and spinal thalamic tract cells, resulting in hyperalgesia [14]. Activation of A1 and A3 receptors decreased cyclic ATP levels, but ATP levels could be increased by activation of A2a and A2b subtypes [15]. A1 could inhibit adenylate cyclase through cGMP-PKG signaling pathways and target multiple proteins regulating different pathways to reduce cytosolic calcium concentration [16]. Whereas, expression of A2a in nociceptive neurons could decrease cAMP and restrain Ca^2+^ channels to relieve pain [17].

In our study, virtual screening was firstly carried out to recognize the small molecule compounds targeting A1 and A2a receptors. MD simulations and MM/GBSA methods were utilized to validate affinity and binding stability of the six molecules. Then, the top six small molecules targeting A1 and A2a receptors were selected as potential agents. Overall, our studies may provide valuable information and new ideals for identifying new agonists or inhibitors targeting A1 and A2a receptors in pain treatment.

## Methods

### Data collection and preparation

The X-ray crystal structures of A1 and A2a receptors were downloaded from the RCSB protein data bank (PDB) (http://www.pdb.org/pdb/home/home.do), and the PDB codes were 5N2S and 2YDV [18]. Additionally, the 6402 natural molecules were collected from the ZINC database (http://zinc.docking.org/catalogs/tcmnp). These natural molecular products were ready to dock 3D format structures of protein and commercially available.

### Virtual screening

We utilized the UCSF DOCK (version 6.8), a program typically used to screen small molecule natural products in large libraries, for predicting potential drug agents [19]. The preparation of docking included elimination of solvent molecules and additions of standard charge and hydrogen atom. A flexible ligand docking method was carried out here. The Dock Prep tool of Chimera program provided the receptor binding sites, when we docked the adenosine receptor with small molecule compounds [20]. To avoid the false positives produced by virtual screening, the candidate ligands were docked into A1R and A2aR for subsequent screening. The program Autodock was utilized to complete the automatic docking simulation [21]. Amber scoring approaches were further employed to show the better flexibility [22]. It was prevalent to allow small molecules and proteins to rearrange the structure of the active region for flexible docking [23]. The 2D and 3D structures of the receptor ligands were simulated by Discover Studio (3.0).

### MD simulations

GROMACS 5.1.4 package to complete the MD simulations analysis of A1R/A2aR-ligands was further employed. The antechamber was adopted in AmberTools9, to generate the topological parameters of small molecular natural products. Then, we applied pdb2gmx in GROMACS software package to convert protein topology files [24]. Furthermore, the MD simulation employed the Amber ff99SB force field and the TIP3P water molecule model. AM1-BCC was used for charging. Eventually, we preferred ACPYPE tool to convert into a topology file suitable for GROMACS. MD simulations were employed by a periodic box in the shape of a dodecahedron. The entire simulation process was limited to periodic boundary conditions. The minimum distance between the protein in the box and the edge of the box was 10 Å [25]. We added TIP3P water molecules to simulate water solvents in the box. Additionally, we added a certain amount of Na^+^ and Cl^−^ to make the solution reach a neutral environment of 0.15 M NaCl. To begin with the MD simulations, we utilized the steepest descent algorithm to minimize the energy of the atoms in the entire environment, so the maximum interaction force between the atoms reached 1000 kcal·mol^−1^·nm^−1^. After restricting the positions of the protein and the ligand respectively, we performed 100 ps NVT (constant number of particles, volume and temperature, constant product balance) and 100 ps NPT (constant number of particles, pressure and temperature, constant pressure balance) to balance simulation [26]. Subsequently, under constant temperature (300 K) and constant pressure (1 atm) conditions, we performed MD simulations with a step size of 2 fs and a total of 20 ns.

### Binding free energy calculation

We employed the mm_pbsa.pl tool in the Amber 9 software package to calculate the binding free energy (Δ*G*_bind_) of A1 and A2a receptor complexes [27]. We extracted 100 snapshots in the 2 ns trajectory at equilibrium to calculate the MM/GBSA binding free energy. In this paper, the time period for extracting A1R-ligand was 10 ~ 12 ns, and the time period for extracting A2aR-ligand was 16 ~ 18 ns.

Δ*G*_bind_ can be calculated by the following formula: Δ*G*_bind_ = *G*_complex_ − G_protein_ − G_ligand_ = Δ*H* + Δ*G*_sol_ – TΔS = Δ*E*_MM_ + Δ*G*_sol_ − TΔS. Δ*E*_MM_ can be calculated by the method of molecular mechanics, which represents the gas phase energy between the protein and the ligand. Δ*G*_sol_ represents the sum of the free energy of polar dissolution and the free energy of the non-polar part. TΔS represents the change in conformational entropy after ligand binding [28]. Moreover, we chose the crucial residues, revealing the remarkable difference for the average contributions among the selected residues.

## Results

### Docking accuracy

The redocked pose of 2’MeCCPA and SCH58261 almost overlapped with the original crystal ligand-binding orientation. The 2’MeCCPA formed one hydrogen bond with the Asn1359 in the crystal structure. The SCH58261 generated six hydrogen bond interaction with Thr88, Glu169, Asn253, Ser277, and His 278. It showed the validity of the docking parameters and approaches employed to represent in the crystal structure.

### Virtual screening for A1 and A2a receptors

Six thousand and four hundred two small molecular natural products were virtual screened from ZINC database to dock into A1R and A2aR. Amber score is the notable feature to show the active region structure of small molecules and proteins, which could also be understood roughly as the binding free energy of small molecule protein complexes. The lower score means the better affinity. Firstly, we screened the top 10 small molecular natural products according to amber scores. The results were shown in Table 1 and Table 2. Table 1 showed that the small molecule ZINC19943625 had the highest affinity with A1R, and its amber score was -67.31 kcal·mol^−1^. The small molecule complexes A1R-ZINC19797529 had the lowest affinity, and its amber score was − 52.33 kcal·mol^−1^. The amber score of A1R-ZINC13555217 was − 59.66 kcal·mol^−1^ and A1R-ZINC15989794 was − 59.28 kcal·mol^−1^. Hence, it was speculated that the approximate score may be related to their similar molecular structures. The amber score of A2aR complexes ranged from − 52.31 to − 48.37 kcal·mol^−1^. The small molecule A2aR-ZINC19370372 had the highest affinity, and the small molecule A2aR-ZINC19774391 had the lowest affinity. The results were showed in Table 2. In order to further investigate the combination of A1R and A2aR with small molecules, MD simulations were performed for further study. Consequently, small molecule complexes A1R-ZINC19943625/ZINC02086848/ZINC13555217/ZINC15989794/ZINC04698406 and A2aR-ZINC19370372/ZINC20176051/ZINC57263068/ZINC22790008/ZINC20228412 were selected for further MD simulation analysis.

**Table 1:**
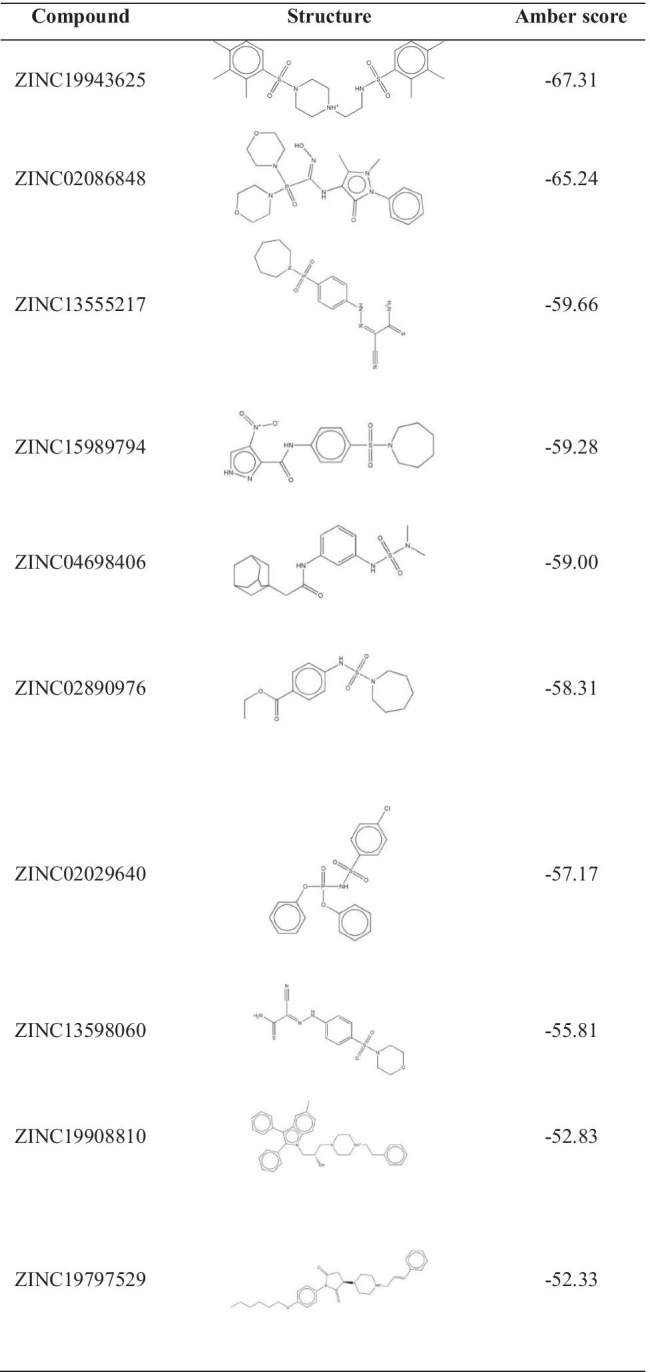
The structure information and amber score of A1 adenosine receptor-ligand complexes

**Table 2:**
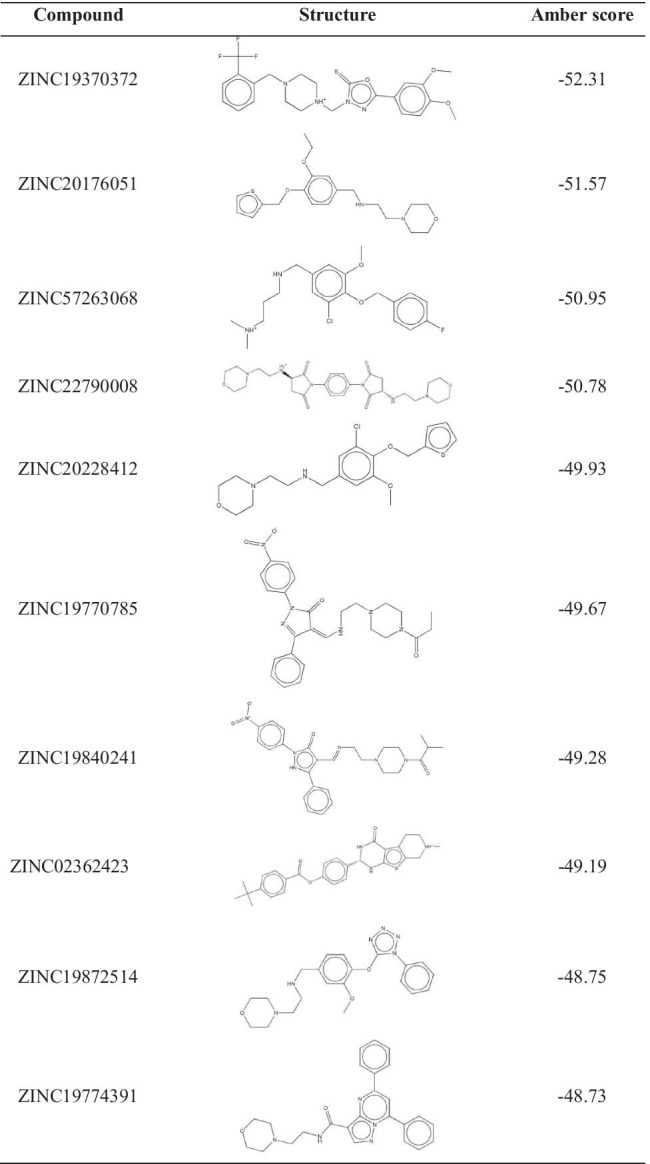
The structure information and amber score of A2a adenosine receptor- ligand complexes

**Fig. 1:**
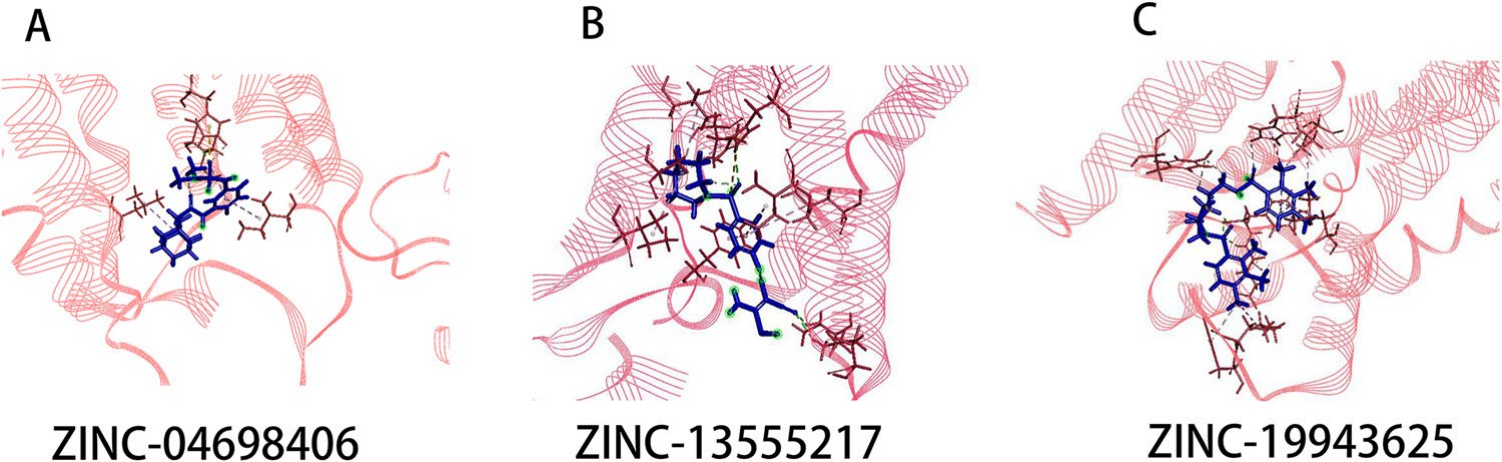
HYPERLINK "sps:id::fig1||locator::gr1||MediaObject::0" 3D structure of the docking small molecule with A1R in the crystallographic complex

**Fig. 2:**
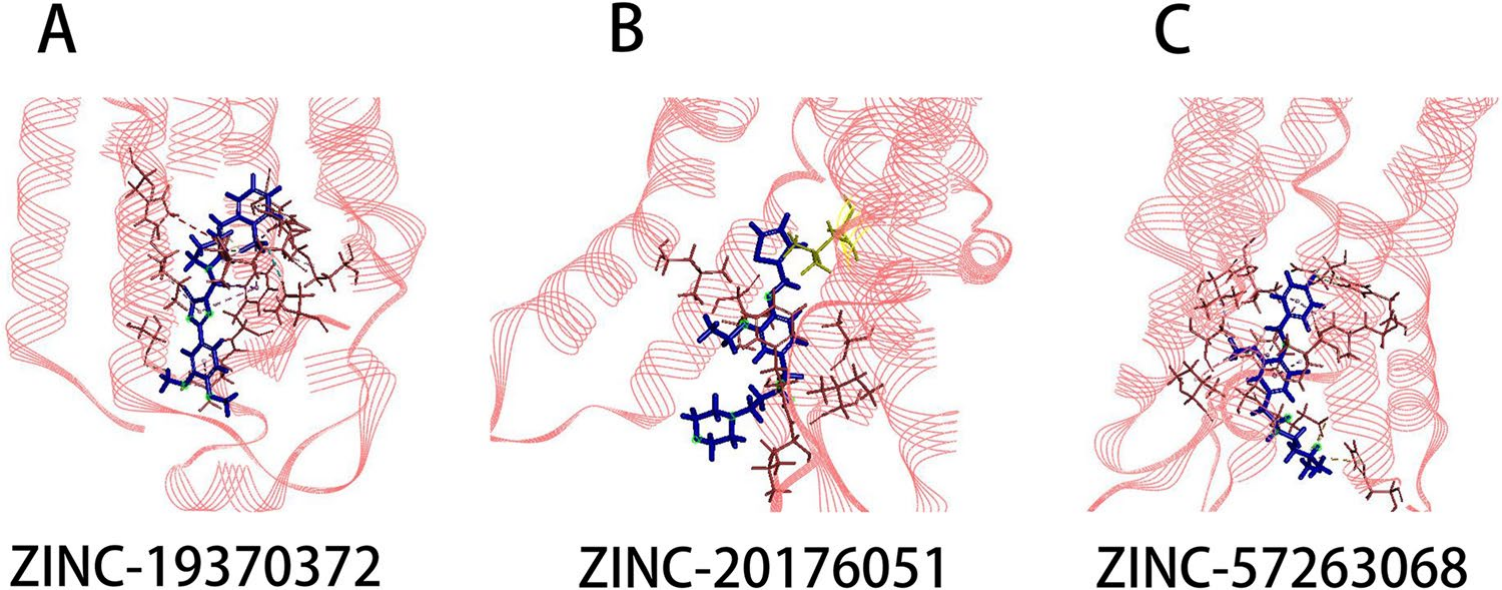
3D structure of the docking small molecule with A2aR in the crystallographic complex

**Fig. 3:**
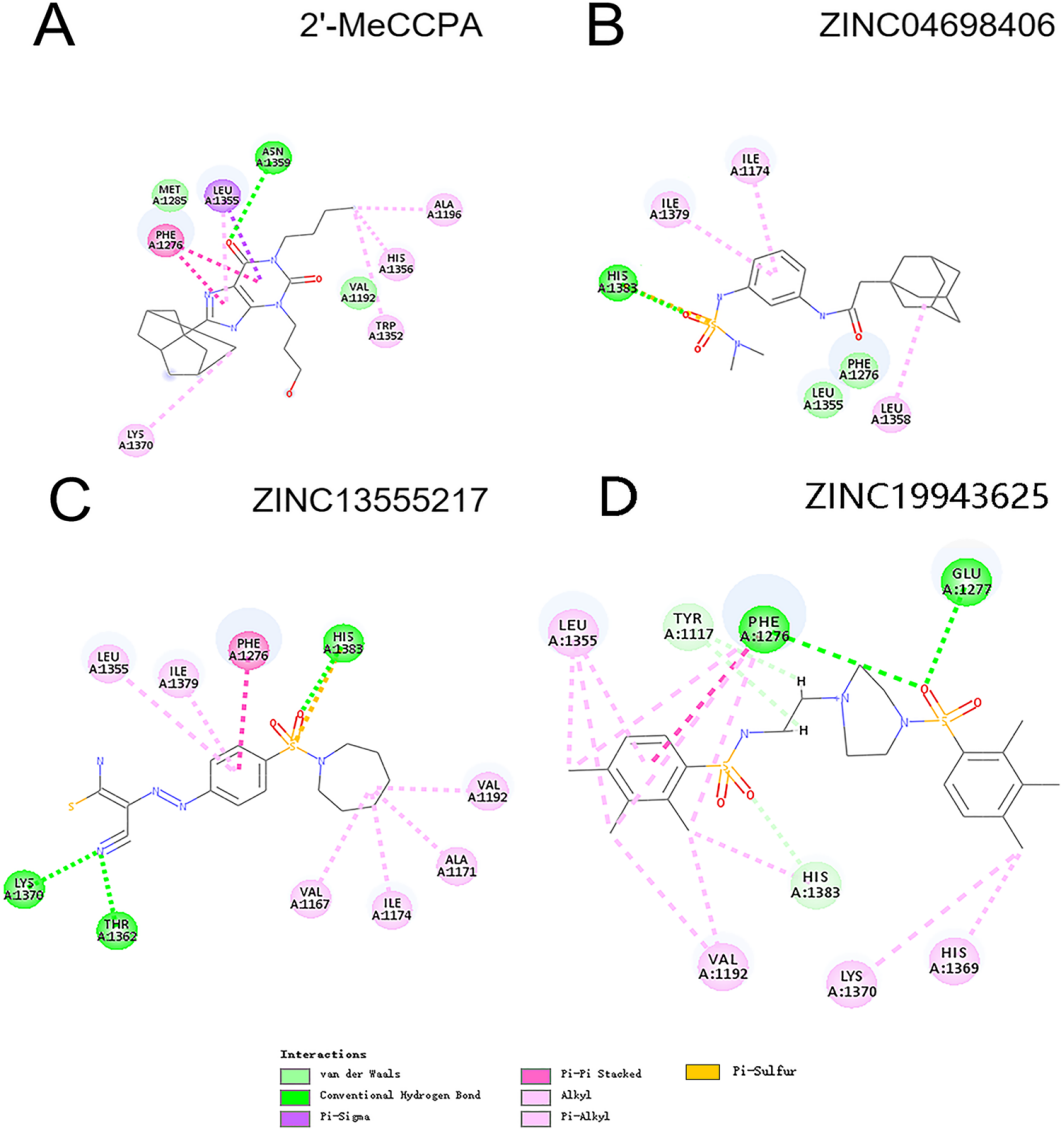
The 2D structure of receptor-ligand interaction of molecule with A1R

**Fig. 4:**
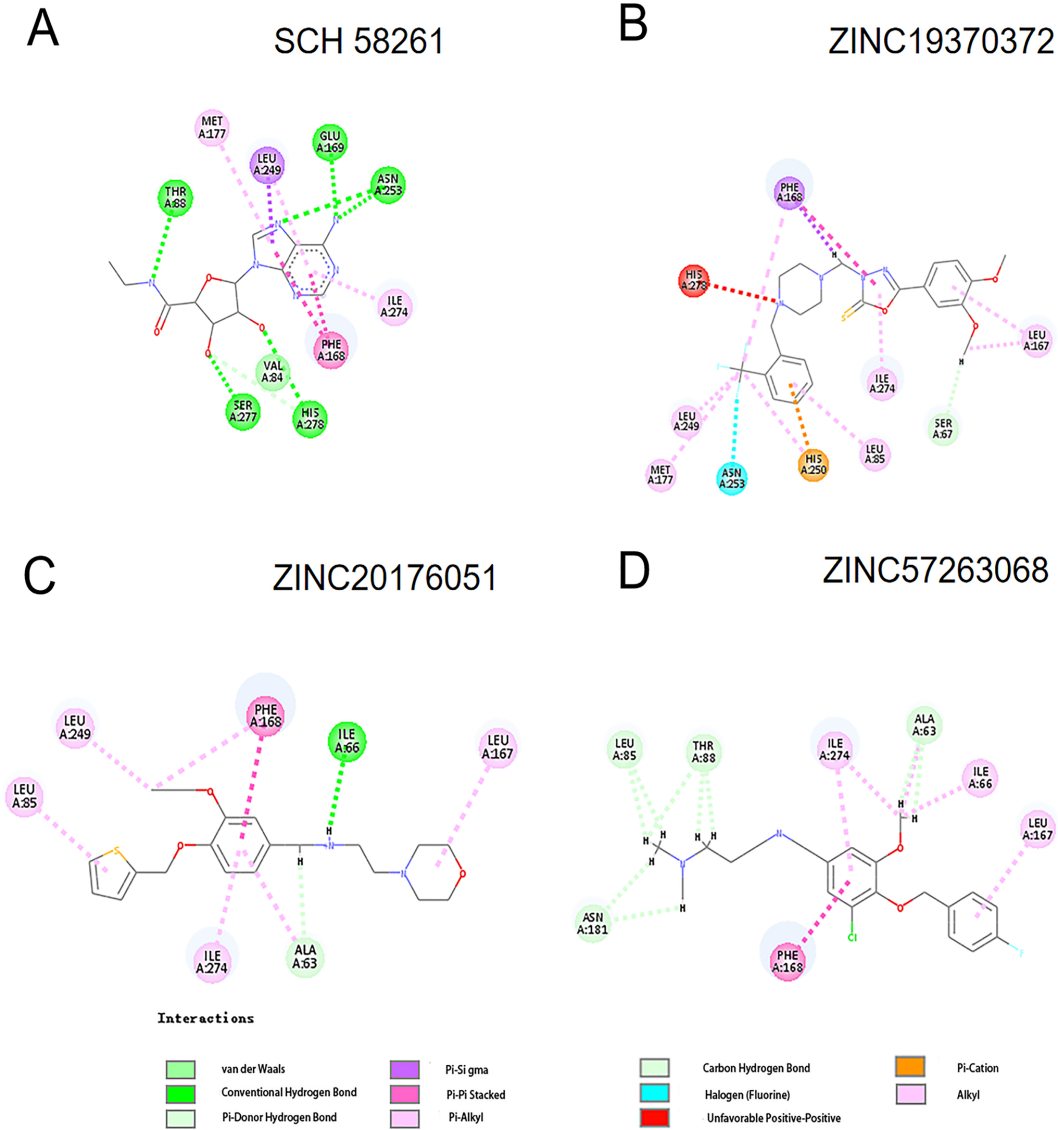
The 2D structure of receptor-ligand interaction of molecule with A2aR

Figure 1 and Fig. 2 showed the ligands had better bind affinity with the receptors, demonstrating the reliability of the docking. The 2D structure could show the residue binding situation (Fig. 3 and Fig. 4). In the reference system, it was shown that there was one hydrogen bond formed between the 2′-MeCCPA and crystal. In A1R-ZINC13555217 binding system, the three hydrogen bonds were formed with bond distance of 2.54A, 2.39A, and 2.42A, involving residues His1383, Thr1362, and Lys1370. And other key residues took part in hydrophobic interactions. As for A1R-ZINC19943625 complex, Phe1276 and Glu1277 participated the building of hydrogen bonds with distance of 2.08A and 2.50A. In ZINC04698406 binding system, residues His1383 formed one hydrogen bond with molecule. Compound A2aR-ZINC57263068 did not formed hydrogen bonds with residues. Maybe it is the reason that why ZINC57263068 has the lowest amber score among the three molecules. For ZINC20176051 binding system, molecule interacted with amido acids of Ile66 via hydrogen bond, and the distance was 2.15A. π-π stacking with benzene ring of Phe168 was formed. Moreover, A2aR-ZINC19370372 complex was found to embed into the active pocket.

### MD simulation analysis

**Fig. 5:**
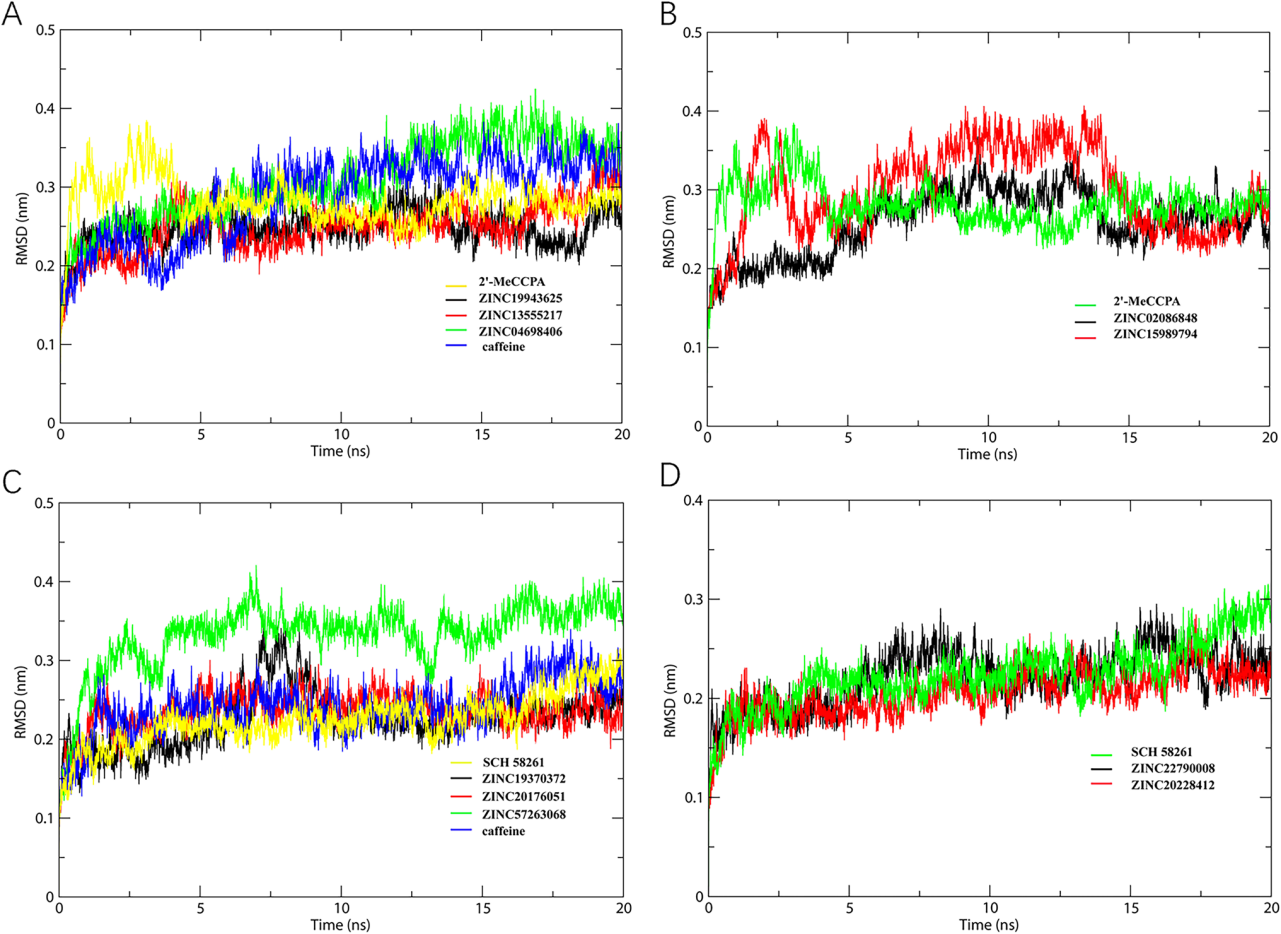
The RMSD results of A1 adenosine receptor-ligand complexes with the pain agonist (**A**–**B**) and the A2a adenosine receptor-ligand complexes with the pain antagonist (**C**–**D**)

**Fig. 6:**
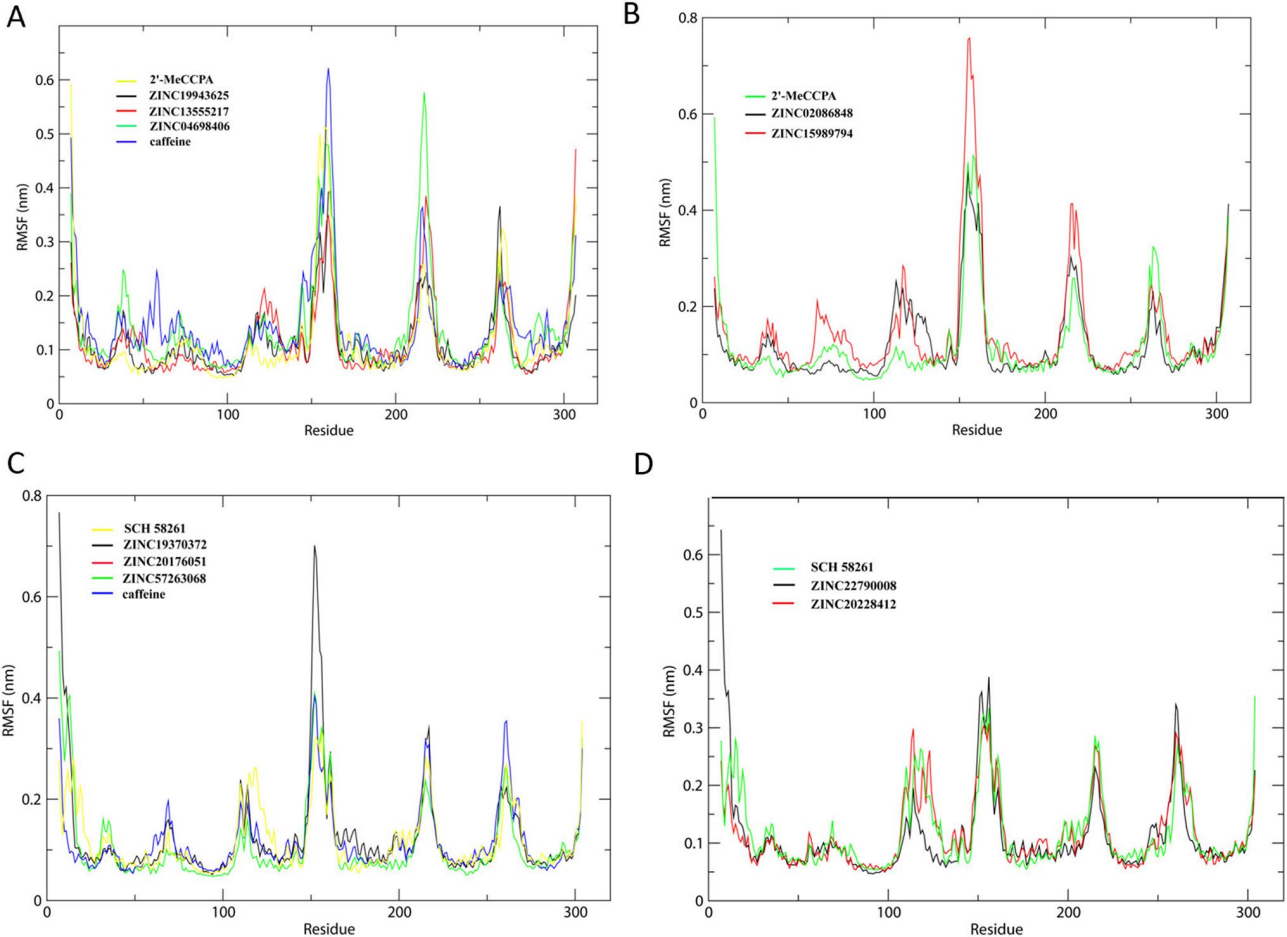
The RMSF results of A1 adenosine receptor-ligand complexes with the pain agonist (**A**–**B**), and the A2a adenosine receptor-ligand complexes with the pain antagonist (**C**–**D**)

We performed 20 ns MD simulations on the 10 small molecular products, and further observed the stability of their binding affinity. RMSD is an important reference data for evaluating the stability of small molecular complexes. The RMSD results of the 10 complexes of A1 and A2a receptors fluctuated between 0.1 and 0.4 nm, indicating that their binding was relatively stable. RMSD of the complexes formed by A1R-ZINC19943625, A1R-ZINC02086848, A1R-ZINC13555217, and A1R-ZINC04698406 were in equilibrium from 0 to 20 ns (Fig. 5). However, the result of the A1R-ZINC15989794 complex was 20 ns, occurring large fluctuations in the MD simulations. Therefore, we speculated that the complexes formed by A1R-ZINC19943625, A1R-ZINC02086848, A1R-ZINC13555217, and A1R-ZINC04698406 were more stable than the A1R-ZINC15989794 complex. ZINC-19370372 remained stable after 10 ns; it could be docked into A2aR with the RMSD value from 0.15 to 0.33 nm. The RMSD value of ZINC-57263068 fluctuated from 0.10 to 0.41 nm. Furthermore, we calculated RMSF to compare the flexibility of various amino acids in small molecular complexes (Fig. 6). In general, the complexes formed by A1R and A2aR with 10 small molecules had similar RMSF results, and the fluctuation range was relatively stable. For A1 receptor-ligand complexes, ZINC04698406 showed the highest value of RMSF during the 100–200 ns. Moreover, the value of ZINC15989794 is higher from 100 to 200 ns. In the A2a receptor-ligand systems, ZINC19370372 had the highest value of RMSF from 100 to 200 ns, suggesting the most increased flexibility. It may correlate with the highest amber score of ZINC19370372.

### Binding free energy and important residues

MM/GBSA was utilized to examine interaction of A1R-ligands and A2aR-ligands. The results of binding free energy and energy components of each complex were listed in Table 3. It was benefit to discover that the binding free energies of A1R-ZINC1994362, A1R-ZINC13555217, and A1R-ZINC04698406 were -109.652 kcal·mol − ^1^, − 1273.594 kcal·mol − ^1^, and − 1351.383 kcal·mol^−1^. Furthermore, the binding free energies of A2aR-ZINC19370372, A2aR-ZINC20176051, and A2aR-ZINC57263068 were − 173.816 kcal·mol^−1^, − 64.789 kcal·mol^−1^, and -132.703 kcal·mol^−1^. Above results revealed a promising binding affinity.

**Table 3:** Binding free energy (kJ/mol) of A1R and A2aR small molecule complexes and their original ligands

Name	Components								
	△*E*_vdw_		△*E*_ele_	△*G*_ploar_			△*G*_nonploar_		△*G*_bind_
SCH 58,261	-176.005 ± 12.125		-71.050 ± 49.476	202.748 ± 26.785			-19.537 ± 0.898		-63.844 ± 27.818
2'-MeCCPA	-138.986 ± 12.302		-109.398 ± 15.230	108.993 ± 10.489			-14.062 ± 1.051		-153.454 ± 14.557
A1R-ZINC19370372	-236.177 ± 12.675		-28.013 ± 13.155	115.605 ± 6.905			-25.231 ± 1.073		-173.816 ± 14.065
A1R-ZINC20176051	-177.154 ± 8.989		-46.368 ± 20.420	179.179 ± 12.803			-20.447 ± 1.176		-64.789 ± 18.073
A1R-ZINC57263068	-186.301 ± 6.708		-88.199 ± 60.429	162.954 ± 32.069			**-**21.156 ± 0.591		-132.703 ± 31.611
A2aR-ZINC19943625	-124.08 ± 16.675	-111.542 ± 30.902			141.292 ± 23.836	-15.314 ± 1.693		-109.652 ± 24.066	
A2aR-ZINC13555217	-158.617 ± 12.118	-1456.554 ± 43.117			359.039 ± 21.891	-17.418 ± 0.954		-1273.549 ± 31.093	
A2aR-ZINC04698406	-134.333 ± 10.531	-1618.546 ± 40.210			416.901 ± 18.543	-15.405 ± 0.773		-1351.383 ± 30.783	

**Fig. 7:**
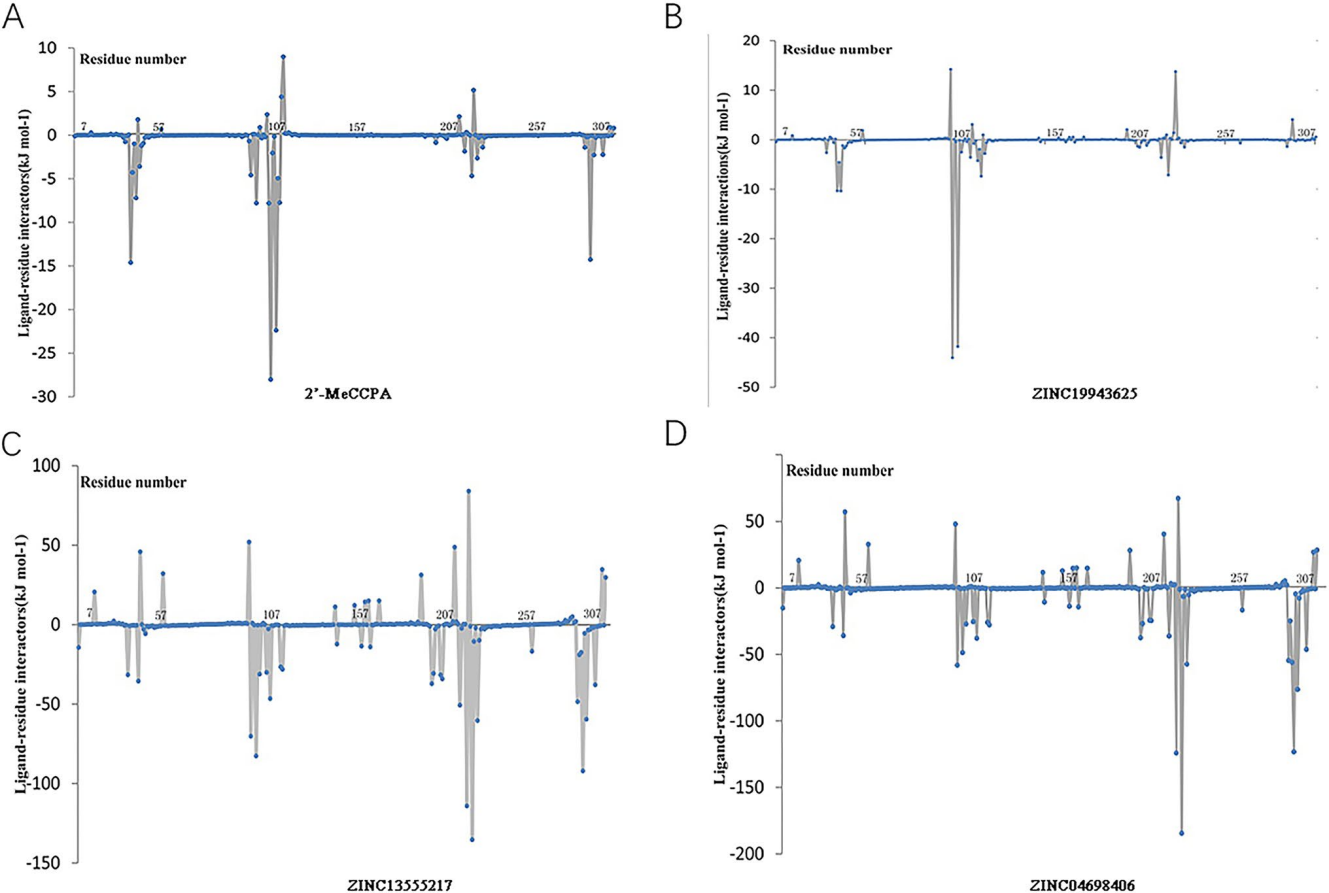
Binding free energy decomposition on per residue of A1 adenosine receptor-ligand complex

**Fig. 8:**
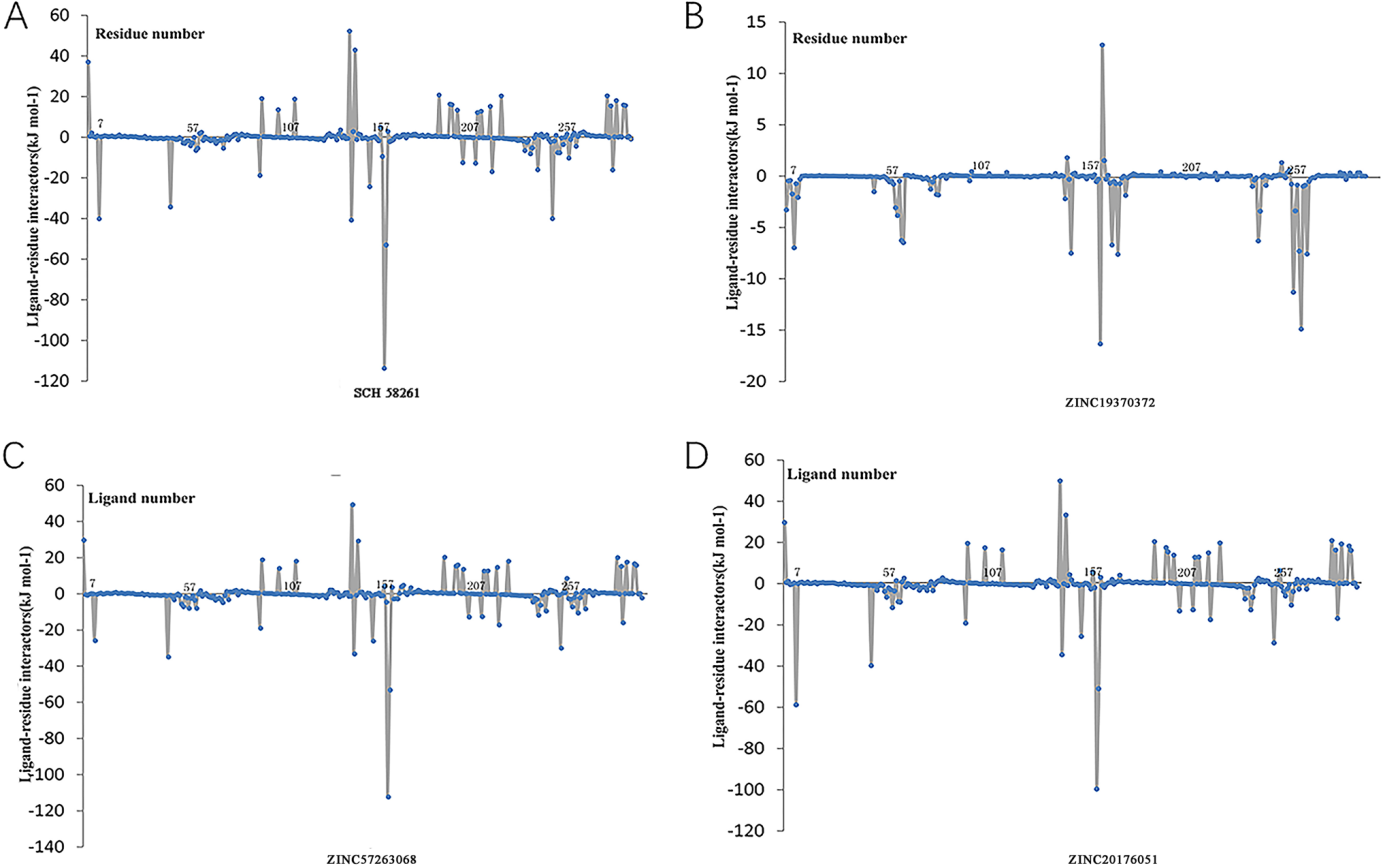
Binding free energy decomposition on per residue of A2a adenosine receptor-ligand complexes

To give full consideration about the surrounding residues and their contribution to the system, we performed binding free energy decomposition, which was based on MM/GBSA method. The results of contribution from residue were shown in Fig. 7 and Fig. 8. Above all, the results indicated that the value of A1R-ZINC19943625 and A1R-ZINC13555217 were close to the 2′-MeCCPA activator. It was obvious to find the three small molecular complexes have the potential to be developed as A1R agonists. Furthermore, the A2aR-ZINC20176051 and A2aR-ZINC57263068 have the analogous binding free energy decomposition like SCH 58,261.

## Discussion

The selectivity and independent potency could be increased by additional structural modification of adenosine [29]. Purinergic signaling has been considered as the interesting ideal for developing new indications in A1 and A2a receptors [30]. Various secondary messengers were controlled by activation of purinergic receptors, including cAMP, DAG, IP3, and Ca2 + [31]. Employing agonists of targeting adenosine receptors to increase endogenous production of adenosine showed better applicability [32].

Adenosine agonists and antagonists played a significant role by making interaction with receptors. Previous studies indicated that adenosine A1 and A2a receptors were abundantly present in sensory nerve endings or some adjacent cells, and received mechanical stimulation or certain chemical stimulation [33]. Subsequently, these simulations were transmitted to sensory neurons, and conducted to the sensory center of the brain along the spinothalamic tract. After discrimination and integration in brain, the stimulation was integrated to form pain sensation [34]. Recent studies indicated that the two serine residues (Ser94 and Ser281) formed hydrogen bonds with the ligand carboxyl group, which were essential for binding of the A1 receptor antagonist [35]. The cAMP levels were reduced by inhibiting adenylate cyclase [36]. Meanwhile, A1 could inhibit Ca^2+^ channels and active K^+^ channels, decreasing Ca^2+^ concentration to reduce the activation of *α*-receptor in the presynaptic membrane of nerves [37].

It was reported that peripheral administration of A2a antagonists could reduce the production of AMP (cAMP) and peptides in sensory neurons [38]. Above results indicated that the changes in G-protein, cAMP, and protein kinase-A were related to the anti-pain function of A2aR antagonists. In functional studies, administration of A2aR agonists may cause mechanical hyperalgesia, anti-inflammatory, and immunosuppressive effects [39]. Moreover, this effect may help indirectly reduce pain signal transduction in the inflamed state.

MD simulation was a kind of computational technique, which can analyze the docking details in biochemical systems [40]. It was significant to utilized drug design approaches for finding the inhibitors of natural products. The approaches combining biochemical and computational studies were utilized to find active regions in receptors [41]. Consequently, we mainly carried out a series of bioinformatics approaches, including virtual screening, MD simulations, MM/GBSA binding free energy calculation, and energy decomposition to discover novel agonist of A1R and antagonist of A2aR. Finally, ZINC19943625, ZINC13555217, and ZINC04698406 were screened to activate A1. They may reduce pain by regulating cGMP-PKG signaling pathway and reducing the activity of adenylate cyclase [42]. The supply of A2aR-ZINC19370372, A2aR-ZINC20176051, and A2aR-ZINC57263068 can reduce the production of the intracellular second messenger cAMP and inactivate the cAMP/PKA/CREB signaling pathway, resulting in degradation of the pain. Together, the six discovered small molecules may not only provide most promising drug candidates for pain patients, but also may help reduce the toxic and side effects of analgesic against pains.

## Conclusion

In this study, a series of in silco approaches were applied to screen the six potential molecules, which could target A1 and A2a. The six candidates might alleviate pian via cGMP-PKG or cAMP/PKA/CREB signaling pathways. Above all, our results would provide novel perspective for further exploring molecular mechanisms in pain, and our promising candidates could be considered as the ideal therapeutic drugs for future pain study.

## Data Availability

The materials, data, and any associated protocols that support the findings of this study are available from the corresponding authors upon request. The molecule data that support the findings of this study are available in http://zinc.docking.org/catalogs/tcmnp. The protein data that support the findings of this study are available in http://www.pdb.org/pdb/home/home.do.
